# 494. Characteristics and Outcomes of COVID-19 in Hospitalized Native American Patients: A Single-Site Retrospective Analysis

**DOI:** 10.1093/ofid/ofab466.693

**Published:** 2021-12-04

**Authors:** T Shaifer Jones, Myles Stone, Amy Nham, Nicole A Bratsch, Trevor N Thompson, Christopher Jentoft, Ryan M Close

**Affiliations:** 1 Whiteriver Service Unit, Indian Health Service, Decatur, Georgia; 2 United States Public Health Service Commissioned Corps, Flagstaff, Arizona; 3 Indian Health Service, Yuma, Arizona; 4 Whiteriver Indian Hospital, Pinetop, Arizona

## Abstract

**Background:**

COVID-19 continues to threaten public health, particularly in Native American (NA) communities, which experienced some of the highest rates of COVID-19 infection and mortality in the US. Although the risk factors and clinical characteristics of COVID-19 are well documented in the general population, there has been little research on NA patients.

**Methods:**

We present descriptive data based on chart reviews of COVID-19 patients hospitalized between April 1 and July 31, 2020 at the Whiteriver Service Unit (WRSU), an Indian Health Service site on the Fort Apache Reservation.

**Results:**

Of the 2,262 COVID-19 cases during the observation period, 490 (22%) were hospitalized and 35 (1.6%) died within 28 days. Compared to previous reports, hospitalized patients at WRSU were younger (median age 54), more likely to be female (55% female), and more likely to have comorbidities (92% at least 1, median 2). Patients under 50 (n=200) often had a history of alcohol abuse (51%) or polysubstance abuse (20%). One third of hospitalized patients (34%) were monitored at home and referred for treatment through a high-risk outreach program. Patients were admitted much earlier at WRSU than in other locations, with a median interval of 4 days from symptom onset to hospitalization compared to 7 days reported elsewhere, but over half were still transferred to higher care. Although WRSU patients had higher rates of comorbidities, the 28-day hospital mortality rate from COVID-19 was nearly half of what has been previously reported (35/490, 7% vs 15-20% reported elsewhere, p < 0.001). This trend persisted after controlling for age. Multivariate logistic regression showed that increasing age, male sex, and high BMI were significantly associated with higher risk of death from COVID-19 (overall model p < 0.001).

Characteristics and outcomes of hospitalized COVID-19 patients at WRSU

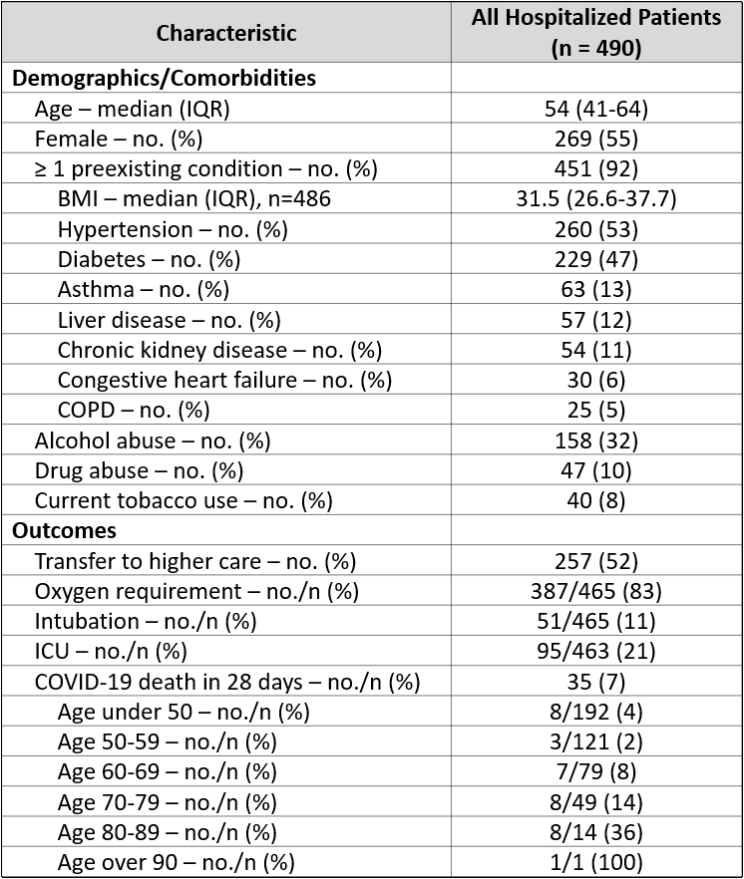

**Conclusion:**

Hospitalized patients at WRSU tended to be younger but with more comorbidities than previous studies. This may reflect the fact that NAs tend to acquire comorbidities at younger ages than the general population. This may also reflect the high rates of substance abuse in younger patients, which could be an additional risk factor for severe COVID-19. We believe that the low mortality rates at WRSU are a result of our outreach program, which likely decreased the interval between symptom onset and medical treatment.

**Disclosures:**

**All Authors**: No reported disclosures

